# The Metabolome of Carbapenem-Resistant Klebsiella pneumoniae Infection in Plasma

**DOI:** 10.1155/2021/7155772

**Published:** 2021-10-22

**Authors:** Zhongwei Wen, Mei Liu, Dong Rui, Xiaoxiao Liao, Rui Su, Zhenming Tang, Zhineng Wen, Zhougui Ling

**Affiliations:** Department of Respiratory and Critical Care Medicine, The Fourth Affiliated Hospital of Guangxi Medical University, No. 156 Heping Road, Liuzhou 545005, Guangxi Province, China

## Abstract

**Aim:**

Carbapenem-resistant *Klebsiella pneumoniae*- (CR-Kp-) mediated infections represent a challenge for clinical practitioners due to their expanding prevalence in hospital environments and antibiotic resistance. However, few studies have shown metabolic changes of carbapenem-resistant *Klebsiella pneumoniae* and CR-Kp-negative patients, and relevant studies are urgently needed.

**Methods:**

In this study, we comprehensively profile the metabolites of 20 CR-Kp-positive and 18 CR-Kp-negative patients in plasma by using 2D gas chromatography–time-of-flight mass spectrometry (GC×GC-TOFMS).

**Results:**

We identified 58 metabolites that were carbapenem-resistant *Klebsiella pneumoniae*-associated. N-Acetyl glucosamine, butanedioic acid, and myoinositol play a significant character in CR-Kp infection.

**Conclusions:**

Our study provides valuable data to serve as potential targets for developing therapies against CR-Kp infection.

## 1. Introduction

*Klebsiella pneumoniae* (K. pneumoniae), a Gram-negative bacterium, is an essential member of the *Klebsiella* genus of *Enterobacterales*. *K. pneumoniae* infections constitute a significant cause of morbidity and mortality globally. These infections generally start elsewhere, typically the lungs, genitourinary tract, or gastrointestinal tract [1, 2].

Carbapenem-resistant *K. pneumoniae* (CR-Kp) is a multidrug-resistant pathogen, which affects people worldwide, prevalent in low, middle, and upper-income countries [3]. The primary mechanism mediates resistance to carbapenems: CR-Kp can produce *β*-lactamases with the ability to hydrolyze cephalosporins and reduce membrane permeability in the cell wall [4, 5]. CR-Kp infections have provided enhanced mortality and many costs for health care systems. Moreover, the pathogen's multidrug resistance to newly expanded antibiotics causes the therapy of *K. pneumoniae* infections much more challenging [6]. Hence, it is urgent to develop and increase our understanding of how host defenses limit CR-Kp diffusion and pathogenesis and produce innovative therapeutic procedures.

Metabolomics (or metabolome) is a rapidly developing method that is aimed at identifying and quantifying the fluctuations in the concentrations of metabolites in a biological sample, such as blood, urine, or saliva [7]. Metabolomics studies are routinely conducted using analytical instrumentation like liquid or gas chromatography-mass spectrometry (LC-MS and GC–MS, respectively) and nuclear magnetic resonance (NMR) to draw a metabolic atlas by detecting metabolites [8, 9, 10, 11]. And this method has been widely used in many studies of Klebsiella pneumonia in the past [12]. Nevertheless, there are few studies on the metabolome of *CR-Kp*-infected patients.

This study is aimed at providing the first comprehensive analysis of the metabolome of CR-Kp-infected patients. Through metabolome analysis of the plasma of infected patients, we can better understand the metabolic changes of CR-Kp infection and discover feasible targets for future treatment.

## 2. Methods

### 2.1. Clinical Samples

Blood samples were collected from 38 patients, which included 20 patients infected by carbapenem-resistant *K. pneumoniae* (CR-Kp) in the Fourth Affiliated Hospital of Guangxi Medical University from March 2016 to July 2020 (Table 1). Blood cultures were performed by automated systems (BacTAlert®, USA). *K. pneumoniae* isolate identity and antimicrobial susceptibility testing (AST) used the Vitek 2 System (bioMérieux, USA). Eighteen adult patients with blood cultures negative for CR-Kp were classified as the CR-Kp-negative group (Table 1). Informed written consent was obtained from each patient. Plasma samples were collected within 24 h after admission. Blood samples were centrifuged at 3,000 × g for 15 minutes at 4°C for plasma collection and deposited at -80°C.

### 2.2. Metabolomics

GC×GC-TOFMS (LECO, USA) sample preparation, derivatization, and spectral acquisition were prepared according to published methods [13]. Briefly, 50 *μ*L of plasma and 300 *μ*L of mixed solvent (methanol : chloroform = 3 : 1, Thermo Fisher, USA) were added into a 1.5 mL centrifuge tube and vortexed for 30 s. Leave the hybrid solution in the refrigerator at −20°C for 10 minutes. After centrifugation at 1000 RPM for ten minutes, transfer 300 *μ*L of the supernatant to the vial and add 10 *μ*L of 0.1 mg mL^−1^ chlorobenzene (Thermo Fisher, USA), alanine (Sigma-Aldrich, USA), and 1 mg mL^−1^ heptadecanoic acid. After the sample was freeze-dried, 80 *μ*L of 15 mg mL^−1^ pyridine (Sigma-Aldrich, USA) dissolved methoxamine and 50 *μ*L BSTFA (1%TMCS, Sigma-Aldrich, USA) were added, respectively. The mixed sample was at 70°C for 60 minutes and then cooled down before injection.

### 2.3. Statistical Analysis

All results are shown as the mean ± SEM. Statistical analysis was conducted using unpaired Student's *t*-tests for two groups and one-way analysis of variance (ANOVA) or two-way ANOVA for multiple groups. Missing values were imputed by MetImp 1.2 [14]. Feature selection and modeling were performed by Random forest, owing to its advantages in small sample sizes, complicated data structure, and high-dimensional feature space. Random forests were implemented using the “randomForest” R package. All analyses were performed using R software (version 4.0.2).

## 3. Results

### 3.1. Metabolic Profiling of Plasma

**Figure 1:**
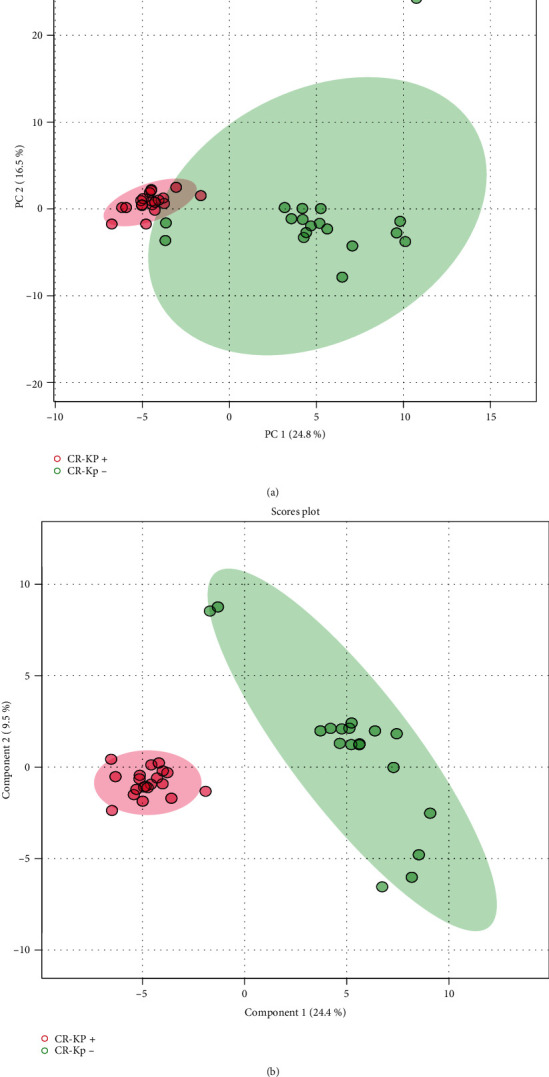
PCA and PLS-DA plot. Based on the metabolic profiling of carbapenem-resistant *K. pneumoniae*- (*CR-Kp*-) infected and CR-Kp-negative patients in plasma.

Principal component analysis (PCA) and partial least squares-discriminant analysis (PLS-DA) analytical procedures were implemented in this investigation to distinguish significantly changed metabolites induced by CR-Kp infection.  Figure 1 shows that the CR-Kp-positive and CR-Kp-negative groups separate in the PCA and PLS-DS plots. This suggests that the significant metabolite variation between the two groups can be identified.

**Figure 2:**
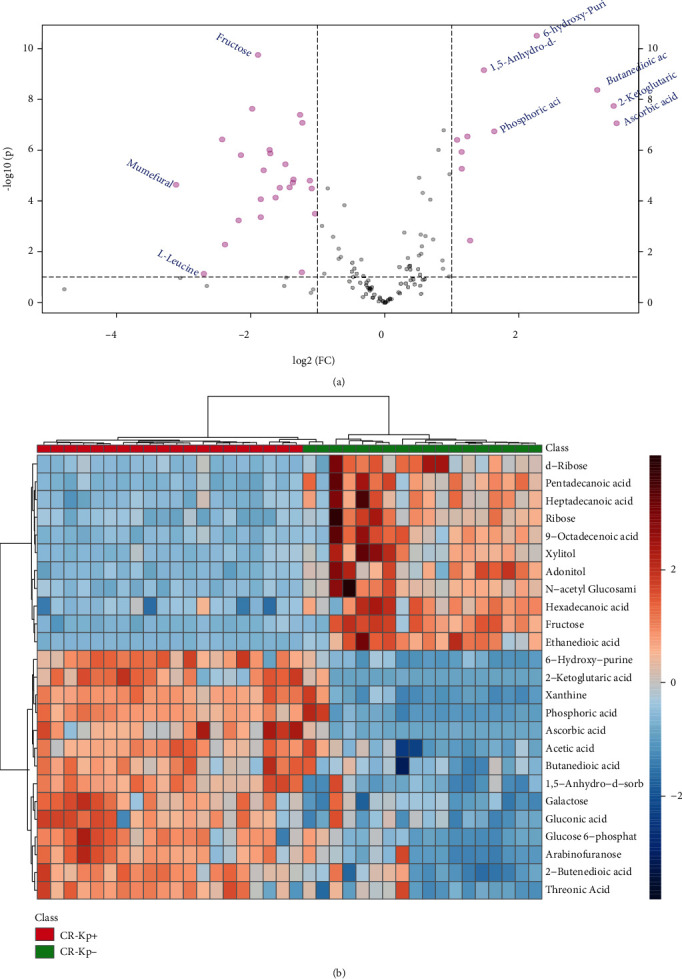
Differential expressed metabolites: (a) volcano plot and (b) heat map of the metabolites in plasma. The outcomes were selected by a threshold of *P* < 0.05.

A total of 58 significantly changed metabolites (*P* < 0.05) were detected (Supplementary Table 1). Carbohydrates, organic acids, fatty acids, and amino acids were significantly altered in the CR-Kp-positive group. Moreover, the volcano plot (Figure 2(a)) indicates differentially expressed metabolites (*P* < 0.05 and ∣log (fold change) | >0.5). In detail, all selected meaningful metabolites are shown in the heat map (Figure 2(b)).

### 3.2. Key Metabolites Identified by Random Forest

**Figure 3:**
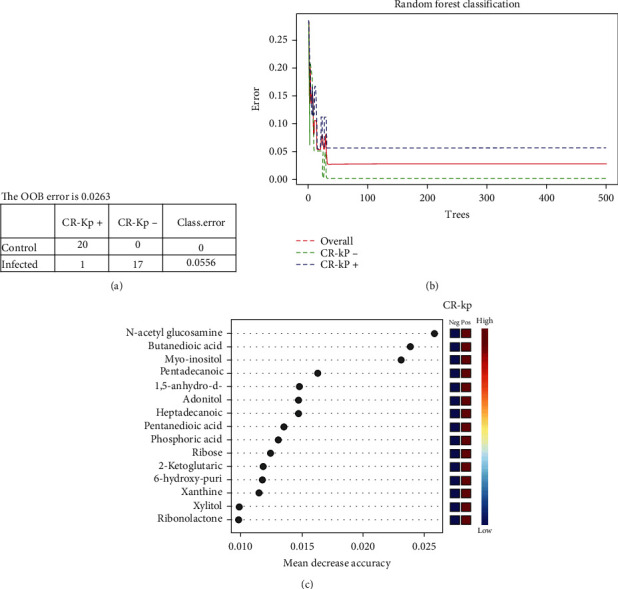
Key metabolites selection: (a) confusion matrix of Random forest classifier; (b) Random forest classification; (c) Random forest variable importance plot. Mean decrease accuracy is the measure of the performance of the model without each metabolite. A higher value indicates the importance of that metabolite in the predicting group (*CR-Kp-*positive vs. CR-Kp negative).

Random forest (RF) was utilized on the dataset for the two comparative cases. The model was built based on 58 significantly changed metabolites.  Figure 3(a) shows the separation between the CR-Kp-negative group and the positive group. RF yielded 98.37% classification accuracy with 100 trees (Figure 3(b)). The variable number dependence section evaluates whether and how much RF performance depends on the number of variables included. This section assesses its capability on an important variable (key metabolites) preference.  Figure 3(c) displays the top 15 metabolites' mean decrease accuracy, revealing how much the model loses by excluding each variable. The more the accuracy endures, the more influential the variable is for the successful classification. The variables are shown with descending importance. N-Acetyl glucosamine, butanedioic acid, and myoinositol are the most significant metabolites with the differential infected and control groups.

### 3.3. Key Metabolite's Function Analysis

Differentially expressed metabolite correlation analysis was applied to analyze the consistency of metabolite trends (Supplementary Figure S1). The metabolite's interrelation was assessed by Pearson correlation, and the results reveal significant metabolite-metabolite correlations. All differential metabolites were mapped to terms in the KEGG database and GO pathway to understand the functions of differential metabolites and the biological processes related to *CR-Kp* infection. The KEGG and GO enrichment analysis showing the top 25 enriched functions is shown in Supplementary Figure S2. Pathway analysis revealed neomycin, kanamycin, and gentamicin biosynthesis as the top canonical pathways (Supplementary Figure S2A). Similarly, the differential metabolites also could be enriched in ammonia recycling, urea cycle, and glutamate metabolism (Supplementary Figure S2B).

## 4. Discussion

Pneumonia remains a preeminent cause of death and hospitalization worldwide, particularly between teenagers and aging individuals [15, 16]. *K. pneumoniae* is one of the most generally isolated pathogens in pneumonia, leading to sepsis [17]. Still, *K. pneumoniae* is a severe threat, and increasing antimicrobial resistance exacerbates this problem [18]. Although diverse *K. pneumoniae* isolates may differ in their resistome, according to geographic area, cultural community switch, and antibiotic stewardship, this species exhibits an amenable ability to concentrate and rearrange resistances [19]. Future metabolic and translational studies must be needed to decipher specific targets to design targeted prevention and treatment.

This study serves the first use of GC×GC-TOFMS for the analysis of the metabolomics profiling of CR-Kp-infected patients in plasma. We found that 58 metabolites were differentially expressed in CR-Kp-positive infected patients' plasma but not the negative ones. Focusing on these metabolites, PCA showed good separation between the samples from both groups. Our study showed that patients infected with CR-Kp had significantly altered metabolic profiles of carbohydrates, organic acids, fatty acids, and amino acids. Notably, N-acetyl glucosamine, butanedioic acid, and myoinositol are the most significantly changed metabolites between the two groups. Research shows that N-acetyl glucosamine (GlcNAc) sugar residues within the core LPS in several species of Gram-negative bacteria play a vital role in targeting the DC-SIGN receptor. DC-SIGN is an innate immune receptor, and the interaction of bacterial core LPS and DC-SIGN may represent an ancient interaction between Gram-negative bacteria and host phagocytic cells [20]. Butanedioic acid (succinic acid) is a dicarboxylic acid in the TCA cycle. Moreover, succinic acid is a microbial metabolite produced by *Escherichia coli*, *Pseudomonas aeruginosa*, and *K. pneumoniae* [21]. Study shows that *K. pneumoniae* utilizes glycerol as the carbon source to produce succinic acid [22]. Blocking the production of succinic acid may be a potential target for inhibiting CR-Kp. Additionally, myoinositol is rarely reported as a newly discovered metabolite in *K. pneumoniae* infection, which may also be a potential growth inhibitory target. The neomycin, kanamycin, and gentamicin biosynthesis pathways were significantly changed in the CR-Kp-positive group. We infer that it is related to the use of *β*-lactam/*β*-lactamase inhibitor combinations.

In conclusion, this study identifies 58 significant CR-Kp-associated molecules in GC×GC-TOFMS. Myoinositol changes significantly in the CR-Kp-positive and CR-Kp-negative groups and can be used as a potential target for treating CR-Kp infection. Future studies are required to better explore those metabolites as a potential therapeutic target.

## 5. Limitations

Our study has some notable limitations. To our knowledge, our study is the first metabolome study of carbapenem-resistant *Klebsiella pneumoniae*. However, it cannot be used by the clinicians directly. Further, the infected patient sample size was limited, and further study is warranted to confirm our findings.

## Figures and Tables

**Table 1:** Demographic and clinical characteristics of patients with carbapenem-resistant K. pneumoniae (CR-Kp) bloodstream infections compared with CR-Kp-negative patients.

Characteristic	CR-kp positive (*n* = 20)	CR-kp negative (*n* = 18)
Age (median, range) (yr)	58 (41-70)	58 (40-70)
Sex		
Male	13	12
Female	7	6
Hospital	20	18
Comorbidity		
Diabetes	6	1
Malignancy	2	0
Chronic kidney disease	2	3
Chronic pulmonary disease	1	1
Antibiotic		
Use of any antibiotic	20	18
*β*-Lactam/*β*-lactamase inhibitor combinations	15	2
Cephalosporins, second generation	2	2
Cephalosporins, third and fourth generations	16	18
Carbapenems	2	10
Mechanical ventilation	5	0
Infection related		
ESBL production^∗^	7	8
VIM-1 production	5	4
MIC of carbapenems^∗∗^		
>4 *μ*g/mL	5	3
≤4 *μ*g/mL	15	15
Final death	5	0

^∗∗^ESBL: extended spectrum beta-lactamase; ^∗∗^the MICs of both imipenem and meropenem were >4 *μ*g/mL.

## Data Availability

Data is available on request. The original contributions presented in the study are included in the article/Supplementary Material. Further inquiries can be directed to the corresponding authors.

## Supplementary Materials

Supplementary Materials Supplementary Figure S1: Spearman correlation coefficient analysis. The value > 0 indicates a positive correlation, while a value < 0 indicates a negative correlation. Supplementary Figure S2: function enrichment analysis. A, KEGG pathway analysis; B, GO pathway enrichment. Supplementary Table 1: carbapenem-resistant Klebsiella pneumoniae infection-associated metabolites.
