# Supplementary material for: The Metabolome of Carbapenem-Resistant Klebsiella pneumoniae Infection in Plasma
Source: Dis Markers. 2021 Oct 22;2021:7155772. doi: 10.1155/2021/7155772 (PMC8556109; doi:10.1155/2021/7155772)
Supplement: Supplementary Materials — Supplementary Figure S1: Spearman correlation coefficient analysis. The value > 0 indicates a positive correlation, while a value < 0 indicates a negative correlation. Supplementary Figure S2: function enrichment analysis. A, KEGG pathway analysis; B, GO pathway enrichment. Supplementary Table 1: carbapenem-resistant Klebsiella pneumoniae infection-associated metabolites. [file 7155772.f1.pdf]

Supplementary Appendix

The metabolome of carbapenem-resistant Klebsiella pneumoniae infection in plasma

Supplementary Figure S1. Spearman correlation coefficient analysis. The value >0 indicates a positive correlation, while a value <0 indicates a negative correlation.

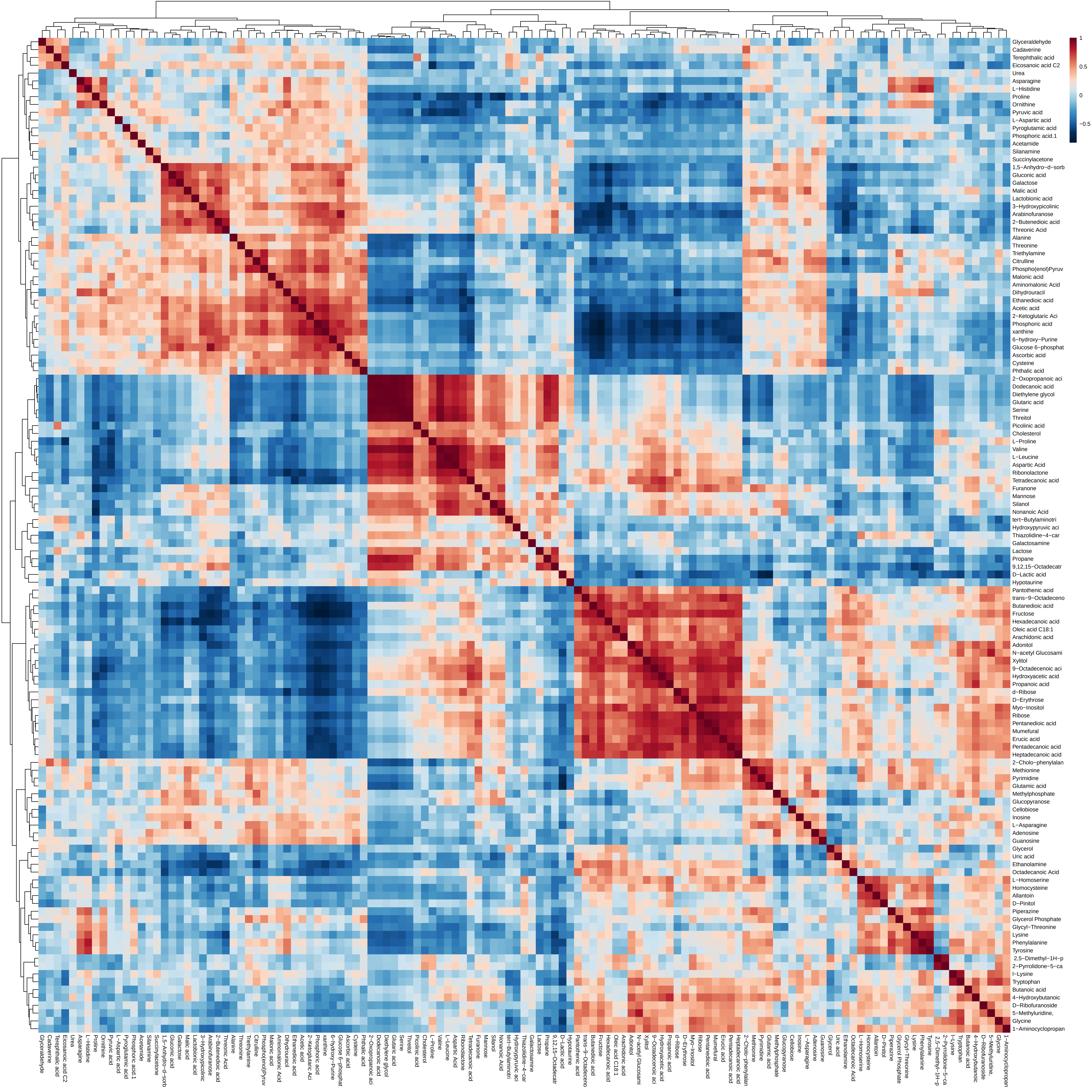

1
0.5
0
−0.5
Glyceraldehyde
Cadaverine
Terephthalic acid
Eicosanoic acid C2
Urea
Asparagine
L−Histidine
Proline
Ornithine
Pyruvic acid
L−Aspartic acid
Pyroglutamic acid
Phosphoric acid.1
Acetamide
Silanamine
Succinylacetone
1,5−Anhydro−d−sorb
Gluconic acid
Galactose
Malic acid
Lactobionic acid
3−Hydroxypicolinic
Arabinofuranose
2−Butenedioic acid
Threonic Acid
Alanine
Threonine
Triethylamine
Citrulline
Phospho(enol)Pyruv
Malonic acid
Aminomalonic Acid
Dihydrouracil
Ethanedioic acid
Acetic acid
2−Ketoglutaric Aci
Phosphoric acid
xanthine
6−hydroxy−Purine
Glucose 6−phosphat
Ascorbic acid
Cysteine
Phthalic acid
2−Oxopropanoic aci
Dodecanoic acid
Diethylene glycol
Glutaric acid
Serine
Threitol
Picolinic acid
Cholesterol
L−Proline
Valine
L−Leucine
Aspartic Acid
Ribonolactone
Tetradecanoic acid
Furanone
Mannose
Silanol
Nonanoic Acid
tert−Butylaminotri
Hydroxypyruvic aci
Thiazolidine−4−car
Galactosamine
Lactose
Propane
9,12,15−Octadecatr
D−Lactic acid
Hypotaurine
Pantothenic acid
trans−9−Octadeceno
Butanedioic acid
Fructose
Hexadecanoic acid
Oleic acid C18:1
Arachidonic acid
Adonitol
N−acetyl Glucosami
Xylitol
9−Octadecenoic aci
Hydroxyacetic acid
Propanoic acid
d−Ribose
D−Erythrose
Myo−Inositol
Ribose
Pentanedioic acid
Mumefural
Erucic acid
Pentadecanoic acid
Heptadecanoic acid
2−Cholo−phenylalan
Methionine
Pyrimidine
Glutamic acid
Methylphosphate
Glucopyranose
Cellobiose
Inosine
L−Asparagine
Adenosine
Guanosine
Glycerol
Uric acid
Ethanolamine
Octadecanoic Acid
L−Homoserine
Homocysteine
Allantoin
D−Pinitol
Piperazine
Glycerol Phosphate
Glycyl−Threonine
Lysine
Phenylalanine
Tyrosine
2,5−Dimethyl−1H−p
2−Pyrrolidone−5−ca
l−Lysine
Tryptophan
Butanoic acid
4−Hydroxybutanoic
D−Ribofuranoside
5−Methyluridine,
Glycine
1−Aminocyclopropan

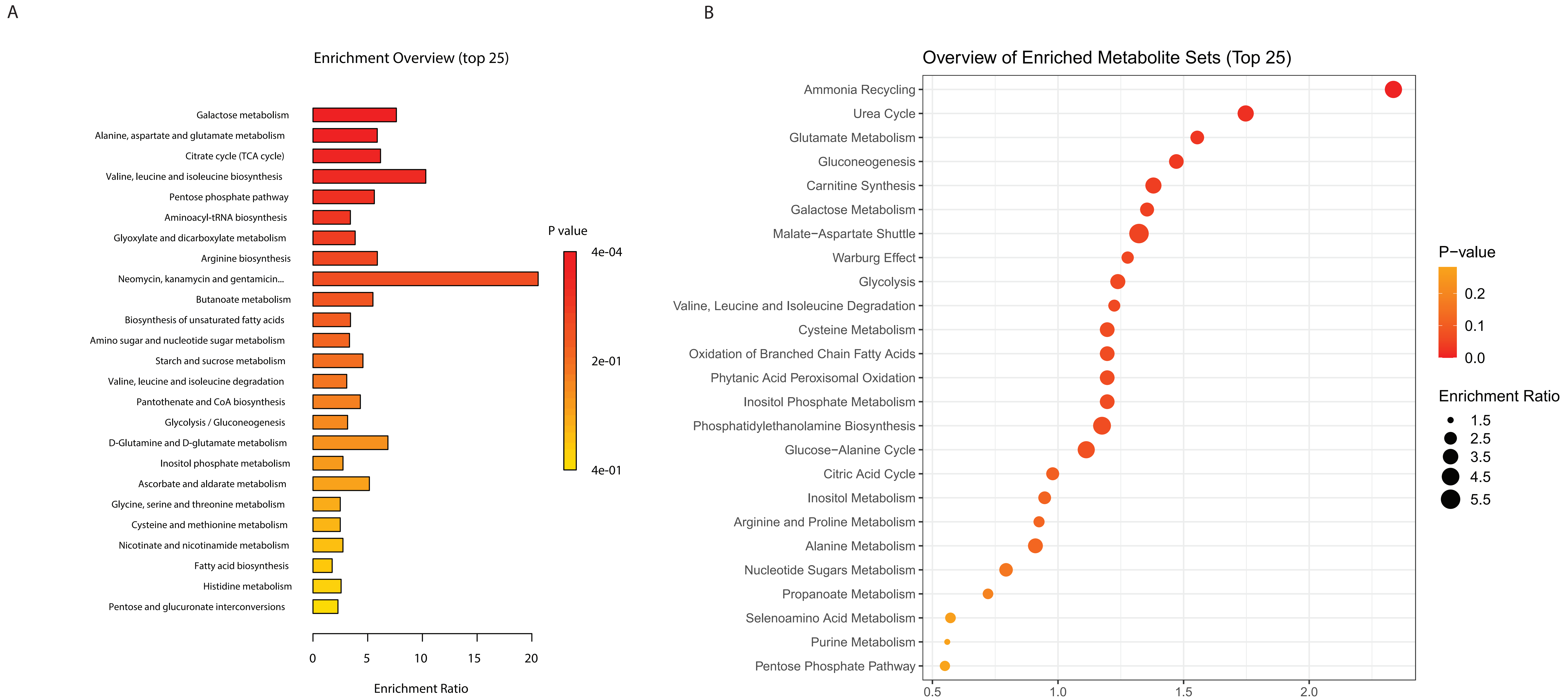

Supplementary Figure S2. Function enrichment analysis. A, KEGG pathway analysis; B, GO pathway enrichment.

Supplementary Table 1. Carbapenem-resistant Klebsiella pneumoniae infection-associated metabolites.

	t.stat	p.value	Log2(Fold Change)	FDR
6-hydroxy-Purine	9.4068	3.10E-11	2.2669	3.94E-09
Fructose	-8.7823	1.77E-10	-1.8892	1.12E-08
xanthine	8.3184	6.66E-10	1.9067	2.24E-08
1,5-Anhydro-d-sorbitol	8.2989	7.05E-10	1.4794	2.24E-08
Butanedioic acid	7.5598	6.14E-09	3.0752	1.56E-07
2-Ketoglutaric Acid	7.1964	1.82E-08	3.4144	3.85E-07
Adonitol	-7.1115	2.35E-08	-1.976	4.26E-07
Pentadecanoic acid C15:0	-6.9303	4.05E-08	-1.2622	6.43E-07
Hexadecanoic acid	-6.6922	8.35E-08	-1.2278	1.11E-06
Ascorbic acid	6.6779	8.72E-08	3.4585	1.11E-06
2-Butenedioic acid	6.4698	1.64E-07	1.2178	1.90E-06
Phosphoric acid	6.4368	1.82E-07	1.6334	1.93E-06
Glucose 6-phosphate	6.2865	2.88E-07	1.2333	2.82E-06
9-Octadecenoic acid	-6.1973	3.79E-07	-2.4238	3.33E-06
Galactose	6.1849	3.94E-07	1.0797	3.33E-06
N-acetyl Glucosamine	-5.89	9.75E-07	-1.7167	7.31E-06
Acetic acid	5.8889	9.78E-07	1.2204	7.31E-06
Arabinofuranose	5.8294	1.17E-06	1.1476	8.29E-06
Heptadecanoic acid C17:0	-5.7833	1.35E-06	-1.7065	9.05E-06
Ribose	-5.7339	1.58E-06	-2.1442	1.00E-05
Xylitol	-5.4661	3.59E-06	-1.4808	2.17E-05
Gluconic acid	5.3334	5.41E-06	1.1495	3.12E-05
d-Ribose	-5.2879	6.22E-06	-1.8046	3.43E-05
Threonic Acid	5.1719	8.88E-06	1.2476	4.70E-05
Ethanedioic acid	5.067	1.22E-05	1.3452	6.22E-05
Erucic acid	-5.0158	1.43E-05	-1.3605	6.99E-05
trans-9-Octadecenoic acid	-4.9821	1.59E-05	-1.1162	7.46E-05
Pentanedioic acid	-4.9212	1.91E-05	-1.3698	8.67E-05
Mumefural	-4.8585	2.31E-05	-3.1114	0.000101
Tetradecanoic acid C14:0	-4.7805	2.93E-05	-1.4179	0.000124
Oleic acid C18:1	-4.7692	3.03E-05	-1.5645	0.000124
Propanoic acid	-4.7505	3.21E-05	-1.5435	0.000125
Hydroxyacetic acid	-4.7477	3.24E-05	-1.088	0.000125
Malic acid	4.6156	4.83E-05	1.178	0.00018
Myo-Inositol	-4.4729	7.43E-05	-1.6269	0.000269
Arachidonic acid	-4.4245	8.59E-05	-1.8481	0.000303
3-Hydroxypicolinic acid	4.4124	8.90E-05	1.154	0.000306
Pantothenic acid	-4.242	0.000148	-1.2481	0.000494
Ethanolamine	-3.9799	0.00032	-1.039	0.001041

Ribonolactone	-3.8719	0.000437	-1.8481	0.001389
D-Erythrose	-3.7709	0.000585	-2.1784	0.001812
Octadecanoic Acid	-3.5934	0.000969	-2.0753	0.002929
Alanine	3.377	0.001771	1.264	0.005229
Dihydrouracil	3.3111	0.002121	1.243	0.006123
Cysteine	3.2603	0.002436	1.133	0.006875
1-Aminocyclopropanecarboxylic acid	-3.2363	0.0026	-1.121	0.007177
Citrulline	3.1469	0.003306	1.112	0.008934
Lactobionic acid	3.1103	0.003645	1.2739	0.009644
5-Methyluridine,	-2.9718	0.00525	-2.3815	0.013608
Phospho(enol)Pyruvic acid	2.9105	0.006155	1.2649	0.015632
Homocysteine	-2.8255	0.007654	-2.3713	0.01906
L-Aspartic acid	2.642	0.012121	1.113	0.029603
Malonic acid	2.5787	0.014152	1.121	0.033912
D-Ribofuranoside	-2.5237	0.016169	-1.3815	0.038026
Threonine	2.4841	0.017779	1.012	0.041053
L-Homoserine	-2.4514	0.019217	-1.001	0.043581
Lysine	-2.4245	0.020478	-0.984	0.045627
Phthalic acid	2.3886	0.022276	0.982	0.048778

*Fold Change ,CR-kp positive/negative; FDR-adjusted was corrected using the Benjamini–Hochberg multiple correction method.
